# Preferred growth orientation and microsegregation behaviors of eutectic in a nickel-based single-crystal superalloy

**DOI:** 10.1088/1468-6996/16/2/025004

**Published:** 2015-03-23

**Authors:** Fu Wang, Dexin Ma, Andreas Bührig-Polaczek

**Affiliations:** Foundry Institute, RWTH Aachen University, D-52072 Aachen, Germany

**Keywords:** crystal growth, orientation, microstructure, segregation

## Abstract

A nickel-based single-crystal superalloy was employed to investigate the preferred growth orientation behavior of the (*γ* + *γ*′) eutectic and the effect of these orientations on the segregation behavior. A novel solidification model for the eutectic island was proposed. At the beginning of the eutectic island’s crystallization, the core directly formed from the liquid by the eutectic reaction, and then preferably grew along [100] direction. The crystallization of the eutectic along [110] always lagged behind that in [100] direction. The eutectic growth in [100] direction terminated on impinging the edge of the dendrites or another eutectic island. The end of the eutectic island’s solidification terminates due to the encroachment of the eutectic liquid/solid interface at the dendrites or another eutectic island in [110] direction. The distribution of the alloying elements depended on the crystalline axis. The degree of the alloying elements’ segregation was lower along [100] than [110] direction with increasing distance from the eutectic island’s center.

## 1. Introduction

To meet the enhanced requirement of the operating temperature of turbines, advanced single-crystal Ni-based superalloys have elevated contents of refractory elements (Ta, W, and Re), which improves their high-temperature performance [1]. However, the refractory elements segregate during crystallization [2]. During solidification, the dendritic *γ* phase is first frozen and is mainly enriched in W and Re, whereas Al, Ta and Ti segregate into the remaining liquid which forms non-equilibrium eutectic islands in the interdendritic regions at the final stage of the solidification. The large (*γ* + *γ*′) eutectic island is undesirable for the as-cast microstructure in the single-crystal components because, to dissolve it, a longer solution treatment time is subsequently required.

Over the past few decades, a number of experimental and numerical modeling studies have been carried out on the solidification sequence of the (*γ* + *γ*′) eutectic island [2–8] as well as on the effect of processing parameters on the eutectic in Ni-based superalloys [9–13]. However, until now no work has been published on the preferential orientation growth behavior of the (*γ* + *γ*′) eutectic. In the present study, the preferred growth orientation behavior of the (*γ* + *γ*′) eutectic island is presented and analyzed in detail. In addition to this, the segregation profile is characterized as a function of its crystallographic orientation.

## 2. Experiments

The material used in this study was Mar-M 247 LC superalloy (Ni-8.21Cr-0.51Mo-9.57 W-9.26Co-0.7Ti-5.62Al-3.18Ta-1.48Hf-0.075C, wt%). Single-crystal cylindrical bars having a height and diameter of 150 and 20 mm, respectively, were solidified by using the selector method in an ALD Vacuum Technologies, Inc. Bridgman furnace. The shell mold was produced by a standard investment casting procedure. The wall thickness of the mold was 7–8 mm. In each shell mold cluster, the bars were assembled around a central rod. To measure the temperature development in the bars, thermocouples were placed at the top positions where the metallographic samples were taken. For the casting experiment, the shell mold cluster was placed on the copper chill plate in the Bridgman furnace and preheated, poured with superalloy melt, and then withdrawn out of the heating zone through the baffle into the cooling zone. In this experiment, a heater and pouring temperature was 1773 K (1500 °C) was used. A withdrawal rate of *V* = 1 mm min^−1^ was applied. The measured temperature gradient was 1.7 K mm^−1^.

After solidification, the shell mold was cooled down in the furnace. When the temperature in the furnace reached under 473 K (200 °C), the vacuum was released and the casting mold was removed. The casting part was then knocked out of the mold and the bars were separated appropriately from the casting cluster. Finally, the bars were sectioned transversely at the top position (perpendicular to the [001] direction) and samples were mounted and polished for microstructural analysis. The microstructures were characterized using optical microscopy and scanning electron microscopy (SEM) after etching with 60 ml C_2_H_5_OH + 40 ml HCl + 2 g (Cu_2_Cl · 2H_2_O). The concentrations of the alloying elements of different microstructures were measured locally using energy dispersive x-ray spectrometer (EDX).

## 3. Results and discussion

**Figure 1.**
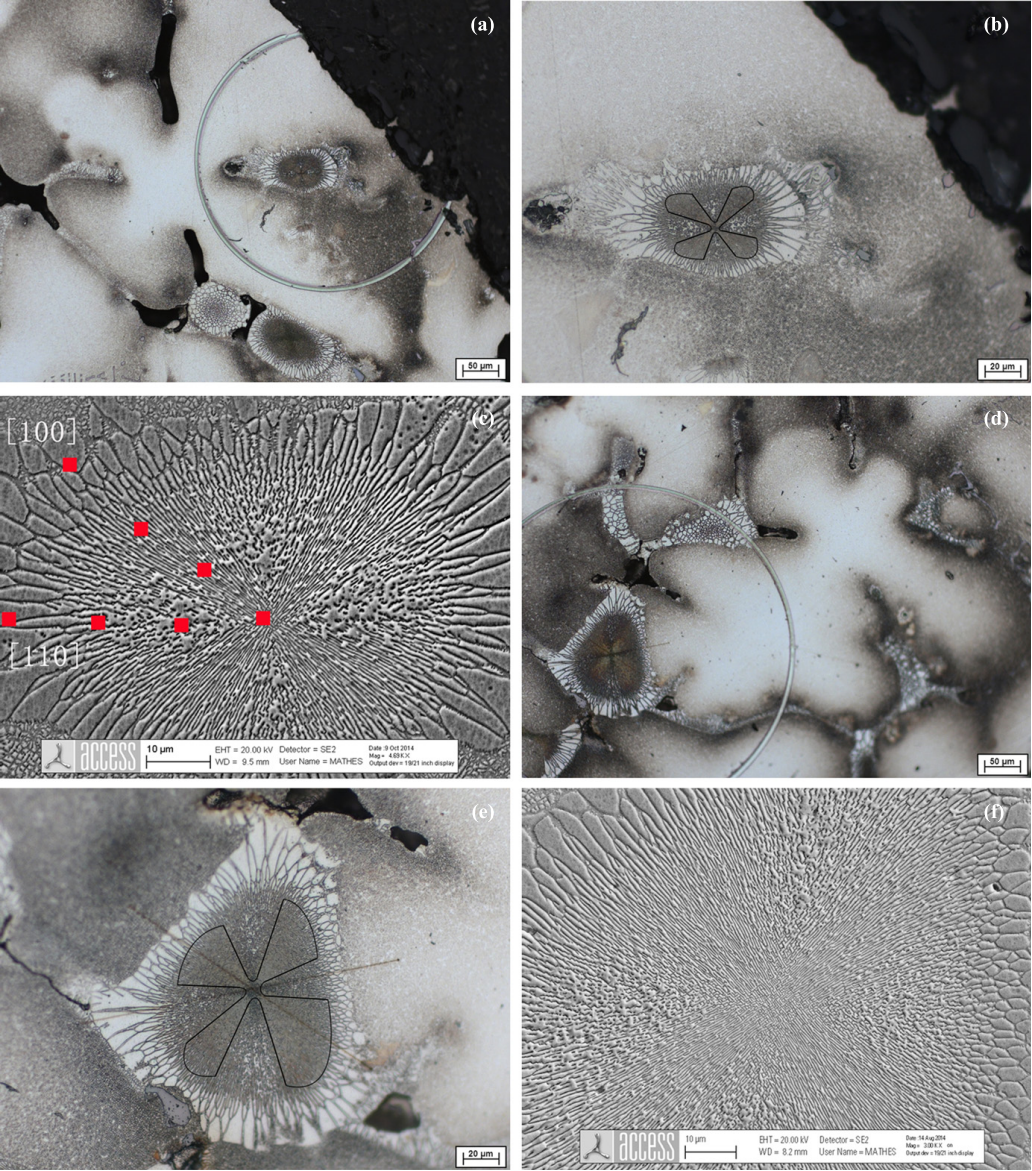
(a) and (d) The morphologies of cruciform dendrites and (*γ* + *γ*′) eutectic island; (b) and (e) magnified photos of the eutectic island; (c) and (f) SEM micrographs of the eutectic island and the measuring paths of the EDX scans along [100] and [110] directions.

Figures 1(a) and (d) show a typical dendritic microstructure and morphologies of the (*γ* + *γ*′) eutectic islands. Since [001] is the preferred direction for the crystal growth in cubic metals and the direction of the primary dendrite arm is [001], the transverse sections of the dendrite are cruciform with orthogonal secondary dendrite arms aligned in [100] and [010] directions. The details of the (*γ* + *γ*′) island shown in figures 1(b) (c) (e) and (f) demonstrate different microstructures depending on the crystallographic orientation.

A regularly arranged (*γ* + *γ*′) microstructure was observed in [100] direction, and *γ* and *γ*′ phases were approximately parallel to [100] direction, whereas a disorderly structure was found in [110] direction. This suggests that anisotropy also exists in the growth of the eutectic. Both *γ* and *γ*′ phases have a face-centered cubic structure and the preferred direction is also [100]. Therefore, an orthogonal cruciform of the transverse sections of the (*γ* + *γ*′) eutectic similar to the dendrite was exhibited in figures 1(b) and (e). The difference in the morphology of the eutectic between [100] and [110] directions depends on the sectioned crystallographic orientation and plane. In this analysis, the sample was sectioned and examined on the (001) plane in which the eutectic lamellar structure along the [100] direction was relatively orderly arranged. In addition to this, it can be seen that the (*γ* + *γ*′) eutectic’s microstructure gradually diverges from the center to the periphery.

**Figure 2.**
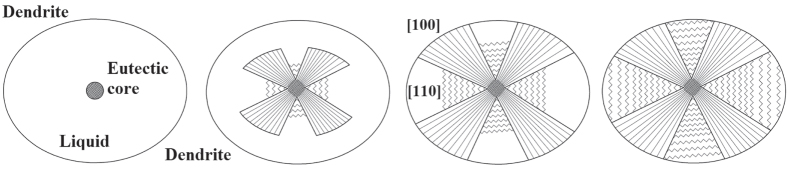
Solidification model of the (*γ* + *γ*′) eutectic island.

**Figure 3.**
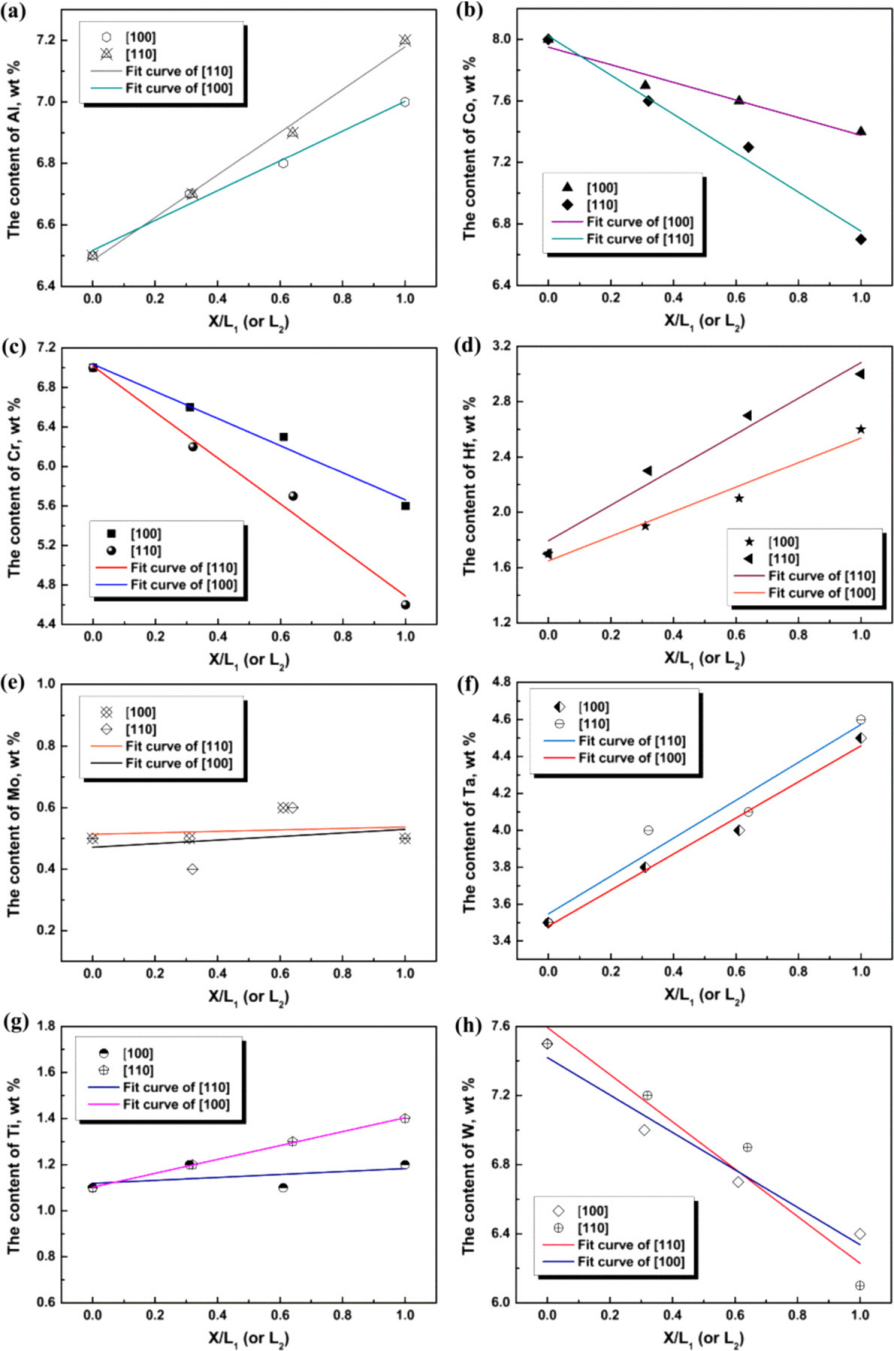
Measured solute distribution from the center of the (*γ* + *γ*′) eutectic island, along [100] and [110] directions.

Based on the examination and the previous studies [6, 14], a novel formation model of the eutectic island can be depicted in figure 2. On commencing, the (*γ* + *γ*′) eutectic core forms directly from the remaining liquid by eutectic reaction. At this stage, the anisotropy has no effect on the formation of the core. As this reaction proceeds, the eutectic prefers to grow in [100] direction, and the advancing velocity of the solidification front is the higher than [110] direction. When the liquid/solid interface of the eutectic in [100] direction impinges on the peripheral dendrites or another eutectic island, and there is no more free space for further growth, the eutectic reaction will cease in this direction, and the eutectic growth continues in [110] direction until the end of solidification. That is, the eutectic growth in [110] direction always lags behind that in [100] direction. Previous studies [15, 16] suggest that a long-range solute diffusion boundary layer established ahead of the eutectic solid/liquid interface may destabilize the morphology of the eutectic interface as a whole. During the solidification of the (*γ* + *γ*′) eutectic, owing to the crystallization of *γ* dendrite around the eutectic, the positive segregation elements such as Al, Ta, Ti and Hf are rejected in the interdendritic remaining liquid, and some of them can enrich ahead of the eutectic interface by a long-distance diffusion. Consequently, a compositional gradient of these elements perpendicular to the interface is built as shown in figure 3. In the present investigation, the longitudinal compositional gradient causes a concave eutectic liquid/solid interface (isotherm) to the remaining liquid. In addition to this, due to the formation of the (*γ* + *γ*′) core, the latent heat is gradually released, which causes a reduction in the nucleating undercooling and retards the eutectic reaction. The *γ*′ and *γ* lamellas have sufficient time to thicken in the final solidification, which will gradually widen the eutectic liquid/solid interface. Since the growth directions of the eutectic lamellas are usually vertical to the isotherm, the eutectic lamella emanates with increasing distance from its center.

As indicted by the red squares in figure 1(c), the concentration of the alloying elements was measured along both [100] and [110] directions in the (*γ* + *γ*′) eutectic island. In figure 2 the measured solute profiles are plotted versus the distance from the eutectic center, along [100] and [110] directions, respectively. According to the sizes in both of the (*γ* + *γ*′) eutectic island’s directions, two different examined lengths (shown in figure 1(c)) were chosen. These were 39.5 *μ*m (*L*_1_) and 40 *μ*m (*L*_2_) for [100] and [110] directions, respectively. To plot the results onto one graph for the same alloying element in both directions, and facilitate the comparison, the center of the eutectic island was set as zero, and the distance *X* to each examined point were then normalized with respect to *L*_1_ or *L*_2_. These dimensionless numbers were used as the abscissa.

Figure 3 shows that the concentrations of Al, Hf, Ta and Ti increase with increasing distance from the eutectic center along [100] and [110] directions, but reduction trends are observed for Co, Cr and W. The concentration of Mo is approximately constant. In addition to this, compared to [110] direction, the concentration gradient of Al, Co, Cr, Hf, W and Ti is smaller in [100] direction. Similar change is found for Ta along the both directions. It indicates that the solute inhomogeneity along [110] direction is more pronounced than that along [100] direction.

In the superalloy system, Al, Ta, Ti, and Hf segregate to the interdendritic regions during solidification, while Co, Cr and W segregate to the dendrite core. Mo does not consistently segregate to either side [17, 18]. At the beginning of solidification, Al, Ta, Ti, and Hf require long-distance diffusion to segregate to the center of the interdendritic region where the eutectic core forms. For this reason, the concentrations of these elements are small in the early crystallized eutectic core. As the solidification proceeds, the eutectic growth approaches the edge of dendrites. A short diffusion distance leads to increasing concentrations of these alloying elements. On the other hand, owing to the thickening of *γ* dendrite, Co, Cr and W are depleted. The concentrations of these elements decrease with increasing distance from the center of the eutectic. In addition to this, due to the lagging eutectic growth in [110] direction, the remaining liquid contains more segregated elements for forming the eutectic in [110] direction. Consequently, the degree of segregation along [100] direction is lower than that along [110] direction. These segregation profiles provide evidence for the preferred growth model of the (*γ* + *γ*′) eutectic islands, as stated above.

## 4. Conclusions

In this paper, a preferred growth orientation behavior of the (*γ* + *γ*′) eutectic and the effect of these orientations on the segregation were investigated. At the beginning of the eutectic’s crystallization, the core directly formed from the liquid by the eutectic reaction, and then preferably grew along [100] direction. The crystallization of the eutectic along [110] always lagged behind that in [100] direction. The eutectic growth in [100] direction terminated on impinging the edge of the dendrites or another eutectic island. Eutectic solidification stopped due to the encroachment of the eutectic liquid/solid interface at the dendrites or another eutectic island in the [110] direction. The degree of the alloying elements’ segregation was lower along [100] direction than that along [110] direction.
